# Teachers’ use of ICT in implementing the competency-based curriculum in Kenyan public primary schools

**DOI:** 10.1186/s42862-021-00012-0

**Published:** 2021-08-23

**Authors:** Julius Murithi, Jin Eun Yoo

**Affiliations:** 1 Teachers Service Commission, Nairobi, Kenya; 2 Korea National University of Education, 250 Taeseongtabyeon-ro, Gangnae-myeon, Heungdeok-gu, Cheongju-si, Chungcheongbuk-do South Korea

**Keywords:** ICT integration, Curriculum implementation, Teacher perception, ICT facilities

## Abstract

The use of Information and Communication Technology (ICT) in education has been widely advocated as much needed 21st-century skills by governments and policymakers. Nevertheless, several challenges in integrating ICT into the curriculum have been reported in previous research, especially in studies on Sub-Saharan African countries. Focusing on the case of Kenyan public primary schools, this study investigated the availability of ICT facilities; teacher capacity to integrate technology into their lessons; and teacher perceptions towards technology in schools. In particular, the study is premised on the constructivist learning theory and the Technology Acceptance Model. A total of 351 teachers completed an online questionnaire. Teachers perceived that ICT facilities were inadequate in schools, which presented a challenge in the integration of technology during the implementation of the new curriculum. Most of the teachers answered that they received only basic computer literacy training. Although teachers perceived the use of computers as necessary, they faced difficulties integrating technology in their lessons. The effect of age and gender on teacher capacity was also investigated in inferential statistics, specifically with Welch tests and Games-Howell post hoc comparisons. Teachers in their 40s had a higher perception of usefulness than teachers in the 30s. Implications of the study are discussed as well as future research topics.

## Introduction

Today more than ever before, the world faces competition in all sectors as a result of the advent of a knowledge-based economy. Governments in all parts of the world are striving to achieve access and good quality education for their citizens (UNESCO, 2013). For this reason, ICT in education is seen as a means of increasing access to education especially to the rural population and making teaching and learning enjoyable. Different studies have supported the use of ICT in education as an enabler in the process of teaching and learning by assisting the learners to grasp concepts that would otherwise have remained abstract (Kozma, 1991). Other scholars contend that the use of ICT in education has little benefit because they are merely delivery mechanisms relying on the teacher’s pedagogical abilities (Clarke, 1983). Amid these debates, policymakers have continued to lay foundations for the use of ICT to profit from the perceived benefits.

Even in developing countries, there have been increased investments in ICTs for schools despite the lack of adequate empirical evidence on the outcomes of such efforts (Piper et al., 2015). However, the Global Innovation Index (GII) 2019 report by the World Intellectual Property Organization ranks South Africa, Kenya, and Mauritius as the leading innovation hubs in Sub-Saharan Africa. This means that there is a need to explore the opportunities and the challenges that exist in these countries about technology and its use in education. In Kenya, the policymakers view ICT in education as an enabler for knowledge acquisition leading to innovation and skill development to address the challenges faced by the country’s education system (Republic of Kenya, 2019). In line with Kenya’s development blueprint, Vision 2030, the education curriculum has been reviewed from the 8-4-4 system to a competency-based curriculum (CBC). The vision of the basic education curriculum reforms is to equip learners with world-class standards and skills needed to thrive in the 21st Century such as digital literacy (KICD, 2017). To achieve this, the integration of ICT in the curriculum is emphasized in the teaching of every subject a shift from the previous system which did not include the integration of ICT in primary schools but only in secondary schools as an elective subject.

Distinctly in the year 2020, education systems in all parts of the world were faced with the challenge of the COVID-19 pandemic. Governments in most countries were forced to close schools and minimize any form of gatherings to contain the spread of the deadly respiratory disease. In Kenya, UNICEF estimated that close to 20 million learners spread across the country were out of school because of COVID-19 (Brown & Otieno, 2020). Therefore, to get a better understanding of whether alternative methods of learning such as e-learning would succeed, this study focused on how teachers and schools were prepared for technology integration before the crisis. The study focused on the assessment of the availability of ICT facilities in public primary schools, teachers' ability to use technology in teaching and learning, and the perception of teachers on the usefulness and the ease of use of ICT. Since digital literacy is considered an important skill to cope with the 21st C developments, the teacher is a crucial player in the successful implementation of ICT and should be well prepared through adequate training (Hwang et al., 2010).

Furthermore, a look at previous studies shows that some challenges have been hindering technology integration in the country. For instance, in a study conducted by Karsenti et al. (2012) in over ten schools around Kenya, various factors were identified as hindrances to the pedagogical integration of ICT. Some of these factors included: lack of ICT devices, the perception of ICT by teachers as time-consuming and as an additional workload, technophobia by older teachers, teachers’ inadequate ICT expertise among others. To address some of the issues, the Jubilee government had a plan in 2013 to integrate ICT in education by providing laptops to all class one pupils (Muinde & Mbataru, 2019). According to Wanzala and Nyamai (2018), by July 2018 19,000 out of 23,951 public primary schools had been provided with technology devices but only 70,000 out of over 300,000 teachers had been trained just months to the rollout of CBC.

A survey by the Teachers Service Commission that purposefully targeted some schools and 1200 respondents also revealed that teachers in public institutions had serious challenges in using ICT in their teaching. 84.2% of the teachers who responded to the survey agreed that they had problems with the use of technology in classrooms. The survey ranked technology integration as the top professional skills gap affecting the delivery of services by teachers (Oduor, 2018; Wanzala & Nyamai, 2018). Therefore, although similar studies have been carried out in the country focusing on the integration of ICT in education, they mostly targeted secondary schools and were done under the 8-4-4 curriculum. In the 8-4-4 curriculum ICT integration was not compulsory in the primary level of education and computer studies were taught as an elective subject in secondary schools. The study was guided by the following three research questions (RQ1 to RQ3):

RQ1. To what extend is ICT infrastructure available in schools to enable the integration of technology in teaching and learning?

RQ2. What is the capacity of teachers to integrate ICT in primary schools in line with the new competency-based curriculum? Are there statistically significant differences in terms of teacher capacity across different age and gender groups?

RQ3. In implementing the new curriculum, what are the perceptions of teachers on the usefulness of ICT, respectively? Are there statistically significant differences in terms of perception on the usefulness across different age and gender groups?

## Literature review

### Theoretical framework

#### Constructivist theory

The constructivist approach is based on the belief that learners can construct and create knowledge from prior experiences in their environment (Kalpana, 2014; KICD, 2017; Waweru, 2018). The proponents of this theory shift the focus from the teacher who was traditionally believed to be the source of knowledge to the learner (Wang, 2008; Waweru, 2018). Two approaches of the constructivist theory were used one targeting teachers' understanding of individual learners and the other that focuses on group learning.

Constructivism can be approached in a way that targets individual learners as well as groups of learners as advanced by Jean Piaget (Kalpana, 2014; Wang, 2008). The theory explains that a learner assimilates new knowledge that adds to an existing body of knowledge. It is therefore important for teachers in the process of integrating ICT to understand that learning can be based on individual discovery and interpretation of information. This realization would help the teacher to emphasize the active participation and involvement of learners to harness their creativity and produce individuals fit for the 21st Century (Kalpana, 2014).

The second approach to the constructivist theory is Vygotsky’s social constructivism that emphasizes collaboration as opposed to individual learning (Waweru, 2018). The proponents of this theory argue that learners grasp concepts better when they work in mixed-ability groups where they share experiences and come up with a common understanding. In such a scenario, the teacher must create a classroom environment that is based on cooperation, democratic principles, and shared creation of content that makes the learners have a sense of ownership of knowledge (Sang et al., 2009). This theoretical understanding was crucial for this study because, in low-resource settings where ICT facilities may not be enough for every learner, the teachers can encourage collaborative learning through device sharing.

#### Technology acceptance model

The Technology Acceptance Model (TAM) is based on the user’s perception of usefulness and the perceived ease of use as cited by Sharples and Modules (2014). The theory has been used widely by researchers in the field of technology in education with various modifications as well as criticism (Bagozzi, 2007). The perceived usefulness of technology relates to the conviction among users such as teachers that it will make their work or that of their learners easier thus enhance job performance (Muinde & Mbataru, 2019). This means that if teachers think that the use of computers would make their day-to-day activities such as preparation of lesson plans, lesson materials, or analyses of student’s results more organized and accurate, then they would probably use them. The perceived ease of use of new or existing technology would mean that the users view technology as one that does not require a lot of effort to learn how to use (Venkatesh et al., 2003). This suggests that teachers would possibly adopt technology that they consider easy to learn and use with minimal need for expert consultation.

Venkatesh et al. (2003) have modified the TAM to include other models in a study that created the Unified Theory of Acceptance and Use of Technology (UTAUT). The study came up with three variables that were thought to directly influence behavioral intention in the use of technology: performance expectancy (perceived usefulness), effort expectancy (perceived ease of use), and social influence. Venkatesh et al. (2003) posit that gender, age, experience, and voluntariness could be classified as moderator variables in the studies on the intention to use technology. They argue that based on socialization, men will prefer to use a certain technology if they perceive that it would help them to accomplish a task. The theory also suggests that the moderating effect of age could be based on the tendency for younger people to be motivated by extrinsic factors such as rewards. We used the moderator variables of age and gender of teachers to compare the differences in perception of the use of technology in education. This was based on the presumed effect of the compulsory use of ICTs in education at the primary level (KICD, 2017) in implementing the new curriculum on the constructs of voluntariness and experience. Therefore, the inclusion of voluntariness in studying a mandatory use system as well as experience in a new system would lead to inconsistencies.

#### Global perception of ICT in education

Globalization and rapid changes in technology have created a knowledge-based economy in the 21st Century. Consequently, governments have invested in the integration of ICT in education at all levels to equip the learners with the skills needed for modern life and beyond (Wambiri & Ndani, 2016). This inclusion and massive investment in educational technology is believed to have had a positive effect in some countries like South Korea where extraordinary economic growth has been experienced since the 1970s (Sanchez et al., 2011).

In addition, Kozma (2003) in a cross-national comparative study of technology and classroom practices involving 28 states posits those different countries such as Taiwan, Finland, the Netherlands, Norway, and Singapore, have had educational reforms to align with global changes. The study adds that the educational reforms in these countries focused on what students learned in school and placed more emphasis on ICT training and interpersonal skills. Various studies have also reported the benefits that technology in education brings to the teachers and learners in different contexts including in developing countries. For instance, Kozma (1991) summarizes his support for the use of technology in education by arguing that different voices and sounds attract the attention of children leading to mental processes that create meaning. Aktaruzzaman et al. (2011) further assert that, when used in the right manner, ICTs in education can bring several benefits such as increased access to education making it more relevant, as well as improving the quality since they make teaching and learning an active process.

The World Wide Web has revolutionized access to information and brought opportunities for e-learning and lifelong learning. Omwenga et al. (2004) argue that this kind of access will not replace the teacher but will provide an opportunity for the learners to meet experts in various fields, researchers, and fellow students. This way they can get firsthand information as well as exchange ideas with their peers from all parts of the world (Redempta, 2012). Hennessy et al. (2010) add that ICTs help in shaping a continued desire for learning that can develop throughout a person’s lifetime, a skill that is needed to survive in a rapidly changing society.

Technology in education also brings a change to the teaching methods used by teachers from the traditional teacher-centered approaches to heuristic styles (Mingaine, 2013a). This change makes classrooms interactive as learners get the opportunity to manipulate technology adding to their creativity and thinking skills needed in the 21st Century (Mwangi & Mutua, 2014). Even in large class size situations where heuristic methods could be difficult to apply, the use of technology can be of great benefit to a teacher in capturing and retaining the attention of learners (Majumdar, 2005).

#### ICT integration in education in Kenya

Kenya like other Sub-Saharan African countries has over the years embedded ICT in its education policies (Mariga et al., 2017; Muinde & Mbataru, 2019). Despite the scarcity of empirical research to show the impact of ICT in learning improvement in the country, the Kenya National Education Sector Plan 2013–2018 focused heavily on ICT integration (Piper et al., 2015). This plan had followed the National ICT policy that was enacted in 2006 to enhance the availability of efficient, affordable, and reliable technology services across all sectors of the economy (Republic of Kenya, 2006).

The value for and recognition of the importance of ICT in education in achieving Kenya’s development blueprint ‘Vision 2030’ led to the provision of tablets to all grade one learners in public primary schools in the country (Langat, 2015; Mariga et al., 2017; Muinde & Mbataru, 2019). This was followed by curriculum reforms aimed at providing every learner in the country with core competencies and world-class digital literacy skills needed to be competitive in the 21st Century (Maluei, 2019).

#### Status of ICT infrastructure in schools

For effective implementation of the policies on ICT in education, there should be adequate infrastructure and facilities. Liang et al. (2005) in a study that draws from 6 years of experience in analyzing the digital classroom environment suggest that some basic facilities are fundamental for ICT integration. They posit that for effective use of technology in education classrooms should be equipped with learner’s devices, teacher’s devices, shared display projectors, network connectivity as well as other enabling installations. This argument is corroborated by Mingaine (2013b) who notes that facilities such as power, computer devices, software, and connectivity are essential for effective ICT integration.

Further, a study by Langat (2015) found out that, infrastructure and ICT equipment shortages were among the challenges facing the implementation of ICT in primary schools in Kenya. The study that targeted 40 primary schools and 450 teachers noted that 94% of the schools did not have ICT equipment, all schools had a shortage of classrooms and only two private schools had functional computer laboratories. Similar challenges were noted in other studies that identified inadequate or limited academic use of computers in primary schools in Kenya as well as a lack of digital customization of classrooms (Tonui et al., 2016; Muinde & Mbataru, 2019).

#### Teacher capacity for ICT integration

Research has demonstrated that ICT in education helps in creating opportunities for the learners to develop 21st Century skills but this depends on the digital literacy of teachers (UNESCO, 2012). Studies on the capacity of teachers in primary schools in Kenya show that, despite the policy formulation for ICT in education and financial investment, the integration of technology in Kenyan classrooms remains low (Piper et al., 2015). For instance, Langat (2015) found that most of the teachers in the study on barriers hindering the implementation of ICT in primary schools in Kenya lacked computer literacy skills. Despite being aware of the importance of technology in education, the teachers blamed the government for the lack of effective planning to offer them in-service training on the use of technology in teaching and learning.

Similar sentiments were made by teachers in a study by Abobo (2018) who asserts two-thirds of the respondents could not integrate technology in the teaching of Kiswahili language. Further, Omolo et al. (2017) also found that student-teachers were able to practice the use of technology in the teaching of Kiswahili in classrooms after learning from their tutors. Both studies suggest that the teachers were willing to apply technology in their teaching after going through training sessions.

However, in some cases where teachers received training, it was basic computer literacy on computer programs such as Microsoft Office and Excel that did not equip them for technology integration in classrooms (Mwangi & Khatete, 2017). Comparably, Wambiri and Ndani (2016) opine that their analysis of documents on primary teacher training in Kenya proved that there was a gap in the pedagogical use of ICT. A study by Muinde and Mbataru (2019) in Machakos County, found that 85% of teachers had received ICT training from the ministry of education. However, 62.3% of the trained teachers felt that the training was not appropriate for teaching and learning. The findings in this study corroborate Majumdar (2005) who observed that most teachers who receive ICT training as part of the professional development (PD) programs still lacked the self-reliance needed to integrate ICT in teaching and learning because in most cases due to time limitations the training only focused on computer applications.

Further, a study to establish teachers’ computer skills in public primary schools was carried out in Homa Bay County by Omito et al. (2019). They used a cross-sectional survey design to collect data from 362 teachers and 85 headteachers. The findings indicated that the number of teachers trained by the government was low, and as argued by Omito et al. (2019) the situation was so since the trained teachers were supposed to train their colleagues. Ngeno et al. (2020) had a similar finding in Ainamoi sub-county that the PD training for teachers did not include all teachers. This study by Ngeno et al. (2020) relates to research by Sharples and Moldeus (2014) that sought to establish the perception of teachers on the readiness for the adoption of technology in public primary schools. The mixed-method case study focused on multi-sites covering different parts of Kenya such as Nairobi, Nakuru, Mandera, and Turkana to compare the integration in both urban and rural areas. Their findings show that only 8% of the teachers felt adequately prepared to use technology in their day-to-day teaching despite 78% of the respondents saying that they perceived computers as easy to use. The study concluded that this difference between the perception of the ease of use and actual use in classrooms was occasioned by poor training on ICT integration.

#### Teacher perceptions on ICT integration

Studies on how perception affects the integration of ICT in education show that what teachers think about the use of technology affects their acceptance and subsequent application in their activities (Wambiri & Ndani, 2016). They argue that the government’s investment through the provision of devices without addressing teachers’ attitudes and beliefs may not yield the desired results. In a study to assess teachers’ beliefs, attitudes, self-efficacy, computer competency, and age, Wambiri and Ndani (2016) found out that younger teachers had a high positive attitude towards technology. This finding they add could be attributed to the younger teachers having received technology training in the teacher training colleges. However, Bebell et al. (2004) observe that teachers’ age and the years of service should be used and interpreted sparingly concerning technology use in schools. They argue that in some specific uses of technology the difference by age would be insignificant if a multi-faceted approach were to be applied in measuring technology usage.

A study on the perception of teachers towards the usefulness of ICT in schools was also conducted by Buliva (2018) in Vihiga County in Western Kenya. The study that used a convenient sample of teachers from the county used the variable of gender to determine whether there were statistically significant differences between male and female teachers. The results obtained from an independent samples *t*-test suggested that there was no statistically significant difference between the mean scores of male teachers. The study concluded that there was no statistically significant difference in perception of the usefulness of computers between the teachers by gender in the County.

While studying the implementation of the laptops project in public primary schools, Muinde and Mbataru (2019) found that 68.5% of the sampled teachers had a high perception of the use of laptops in their teaching and learning. However, they established that 39% of the teachers felt that the time allocated for the integration of technology was not adequate and that most of their lessons were spent assembling the gadgets. In such circumstances, teachers are more likely to resist the use of ICTs in their teaching if they feel that they will spend more time and effort to make them work (Omwenga et al., 2004).

The perception of time and ICT integration was also noted by Heinrich et al. (2020) in a study on the potential and prerequisites of effective tablet integration in rural Kenya. The mixed-method study that involved classroom observation, teacher interviews, student surveys, and focus groups, found that teachers often excluded students perceived to be slow learners during technology integration. Some of the teachers interviewed said that they could not cater to the learners experiencing academic challenges due to the limited time in a lesson. The study recommends more professional development of teachers to equip them with the pedagogical ability to accommodate all learners including those with disabilities in a technology-integrated classroom.

## Methodology

### Participants

Among the population of 1,436 teachers, this study targeted 30% of them (Mugenda & Mugenda, 2003), which was 430. Specifically, convenience and snowball sampling were executed, which was inevitable in the prevailing circumstances occasioned by the global COVID-19 pandemic. By employing snowball sampling, a small number of teachers in the target population responded to the questionnaire and then were asked to assist in reaching out to other prospective participants (Cohen et al., 2018). As teacher gender and age were frequently utilized in previous research, they were put into consideration in sampling. Given that previous research on ICT integration in Kenya has focused on urban areas, more representative sampling incorporating non-urban teachers is warranted (Newby, 2014). Among the 430 sampled teachers, 351 teachers completed the questionnaire with a response rate of 81.6%. The participants were teachers in urban (54.7%) and non-urban (45.3%) areas. They consisted of 4 age groups: 20s (15.1%), 30s (55.3%), 40s (23.6%), and 50s (6.0%). Male teachers comprised 61% of the sample.

## Research instrument and data analysis

A pilot study was conducted to obtain the content validity of the instrument. The process of pre-testing the instrument was done in a neighboring Sub-County outside the area of study but with similar conditions. The respondents were purposively selected from experienced teachers who were asked to comment on the relevance of the content, clarity of the questions, and the time taken to complete the questionnaire. Some items were modified or deleted to accommodate the feedback, which led to the revised questionnaire of 17 items. Frequencies and percentages of the 17 survey items were presented to answer the descriptive part of the three research questions: Items F1 to F6 for RQ1; C1 to C5 for RQ2; and P1 to P6 for RQ3. With regards to the inferential part of the research questions of RQ2 and RQ3, Cronbach’s alphas of the subscales were calculated before proceeding further. The Cronbach’s alpha of all the 17 items was 0.754, but some of the items were removed to increase the internal consistency of the subscales to answer inferential research questions. Specifically, items C1, C2, C4, and C5 had the Cronbach’s alpha of 0.70, and the average of the four items served as the dependent variable of RQ2, teacher capacity for ICT integration. Likewise, the average of P1 and P3 to measure teacher perception on ICT usefulness served as the dependent variable of RQ3, the Cronbach’s alpha of which was 0.66. According to Nunnully (1978), Cronbach’s alpha at or above 0.70 is acceptable as a test for the internal consistency of an instrument. The subscale internal consistency of teacher perception on ICT usefulness was slightly lower but close to the nominal value of 0.70.

For inferential statistics, two-way ANOVAs were initially conducted with gender and age as independent variables for each of the dependent variables. However, Levene’s tests indicated violations of the equal variance assumption. We instead employed the Welch test, a robust statistic used in violations of the equal variance assumption (Welch, 1947). When the Welch test was statistically significant, Games-Howell post hoc tests were conducted for pairwise comparison groups. For the 4 age groups, there were a total of 6 (= 4 combination 2) comparisons per dependent variable.

## Results

### Availability of ICT facilities

The first research question (RQ1) was to investigate the ICT infrastructure availability in public primary schools for the effective implementation of digital learning. The results on the availability of ICT devices are summarized in Table 1. Most of the schools (87.7%) lacked internet connectivity (F1). Approximately 70% of the teachers also answered that their schools did not have projectors as a part of the shared devices essential for the integration of technology in schools (F2). Further, teachers indicated that their schools lacked the customization required for the introduction of digital devices. Specifically, 80% of them answered that their classrooms and computer laboratories did not have sockets and power extension cables (F6) and 73.5% of them also said that they did not have access to the laptops provided by the government (F4). Despite the challenges faced by teachers in accessing devices, 55.8% of the teachers reported that learners had relatively high access to tablet PCs (F5) and 82.9% of them reported reliable power supply (F3).

**Table 1:** Availability of ICT facilities in schools

	Yes		No	
F1: Internet connectivity	12.3		87.7	
F2: Availability projectors	29.3		70.7	
	1	2	3	4
F3: Supply of power is reliable	10.3	72.6	13.1	4.0
F4: Teachers have access to laptops	6.3	20.2	63.8	9.7
F5: Learners have access to tablet PCs	32.2	23.6	31.1	13.1
F6: Classrooms have sockets and extension cables	2.8	17.1	23.9	56.1

1 = strongly agree; 2 = agree; 3 = disagree; 4 = strongly disagree. The numbers indicate percentages

### Teacher capacity for ICT integration

The second research question (RQ2) investigated teachers’ ability to use technology in the performance of their duties (Table 2). Most of the teachers in public primary schools had basic computer skills. The high percentage of teachers with basic computer skills was corroborated by the finding that 77.7% of the respondents had basic computer training as part of their teacher training course. Although many teachers received technology training as part of their pre-service course, we found that there was a challenge in the follow-up in-service training. When asked whether they attended in-service training on technology integration, 66.4% of the teachers disagreed and strongly disagreed; this group of teachers had not participated in any professional development courses to equip them with any relevant pedagogical skills for the application of technology in their lessons. Relatedly, 44.7% of the respondents did not use computers to prepare their instructional materials in preparation for teaching, and 58.4% of the teachers could not plan and integrate technology into their lessons.

**Table 2:** Teachers’ capacity to integrate ICT in teaching

	1	2	3	4
C1: I have basic computer skills	63.0	25.9	7.1	4.0
C2: Computer training was part of my teacher training course	14.5	63.2	14.8	7.4
C3: I Have attended in-service ICT training	8.8	24.8	59.3	7.1
C4: I use ICT for instructional materials	5.7	49.6	24.2	20.5
C5: I can plan and integrate ICT in teaching	24.8	16.8	49.3	9.1

1 = strongly agree; 2 = agree; 3 = disagree; 4 = strongly disagree

### Teacher perceptions on usefulness

Despite the challenges faced by teachers in terms of the availability of facilities and inadequate training, our study demonstrated that teachers had a high perception of technology use (Table 3). The results show that almost all the teachers (98.9%) had the belief that technology would make them more organized and enable student-centered learning to take place in their schools. Further, there was a high belief that the integration of technology would enhance collaboration among learners as shown by 67.5% of the teachers who responded in the affirmative (RQ3). Teachers also had a high attitude towards the usefulness of technology to them as 97.7% of the respondents felt that the integration of technology would make the teachers more organized in their duties. However, the study found that 52.7% of the teachers perceived ICT to be time-consuming and would need more time allocation in the school timetable for successful integration. The findings also suggest that teachers were worried about the learners’ access to the internet as perceived by 60.1% of the teachers who considered it unsafe.

**Table 3:** Teachers’ perception on ICT integration

	1	2	3	4
P1: ICT enhances learner-centered learning	0.3	0.9	32.5	66.4
P2: ICT improves teacher's performance of duties	19.4	78.3	2.3	0
P3: ICT helps learners to collaborate	17.4	50.1	31.9	0.6
P4: Integration requires more time allocation	24.2	28.5	18.5	28.8
P5: The internet is not safe for learners	18.5	41.6	37.0	2.8
P6: Teachers need help from computer experts	45.3	29.6	23.4	1.7

1 = strongly agree; 2 = agree; 3 = disagree; 4 = strongly disagree

### Inferential statistics on the teacher capacity and perceived usefulness

#### The effect of age

Age had a statistically significant effect on the perception of usefulness (RQ3, *p* = 0.000), but had no statistical significance on teacher capacity (RQ2, *p* = 0.059) (Table 4). The Games-Howell post hoc tests indicated that teachers in their 40 s (M = 3.40, SD = 0.34, *n* = 83) had a higher perception of usefulness than those in their 30 s (M = 3.15, SD = 0.36, *n* = 194). Other groups were not statistically different in terms of the perception of usefulness or teacher capacity.

**Table 4:** The effect of age

	W	*df1*	*df2*	*p*		20 s (a; *n* = 53)	30 s (b; *n* = 194)	40 s (c; *n* = 83)	50 s (d; *n* = 21)	G-H
RQ2	2.60	3	71.24	.059		2.97(.33)	2.86(.26)	2.86(.27)	3.01(.40)	N. A
RQ3	10.517	3	71.931	.000		3.27(44)	3.15(.36)	3.40(.34)	3.38(.50)	c > b

*W* Welch statistics. *G-H* Games-Howell. Numbers enclosed in parentheses indicate standard deviations

#### The effect of gender

Both teacher capacity (RQ2) and perceived usefulness (RQ3) were not statistically different by gender (Table 5). Male teachers and female teachers did now show a difference in terms of teacher capacity and perceived usefulness.

**Table 5:** The effect of gender

	W	*df1*	*df2*	*P*	Female (a; *n* = 137)	Male (b; *n* = 214)	G-H
RQ2	.501	1	291.96	.479	2.90(.29)	2.88(.29)	N. A
RQ3	1.377	1	273.98	.242	3.28(.41)	3.23(.38)	N. A

*W* Welch statistics. *G-H* Games-Howell. Numbers enclosed in parentheses indicate standard deviations

## Discussion

Following the importance attached to technology in most parts of the world in almost all sectors, developing countries also have had to make the necessary investments and changes to cope with the 21st Century developments. As a result, education systems have been changed and curricula adjusted to have technology integration in schools. Our study sought to establish the preparedness of Kenyan primary schools for the rollout of mandatory technology use in all subjects of the new curriculum. On infrastructure development, our findings show that shared devices (i.e., projectors, sockets, and extension) cables were not available in most public primary schools. Although access to a computer or laptop by teachers is key in the integration of technology in education (Liang et al., 2005), teachers in most primary schools did not have access to these devices. The findings were consistent with other studies that pointed at the lack of devices for teachers as a threat to technology integration in Kenyan schools (Langat, 2015; Tonui et al., 2016; Mingaine, 2013a, 2013b). This reveals a challenge that has existed over the years despite the significance attached to ICT availability (Langat, 2015; Liang et al., 2005) a situation that calls on stakeholders to prioritize infrastructure installation (Mingaine, 2013a).

On the other hand, learners had relatively high access to technology devices such as tablet PCs. The power supply in schools also appears reliable, which could be attributed to the government’s commitment and investment towards digital learning in public primary schools in the country (Muinde & Mbataru, 2019; Piper et al., 2015). Since not all schools had a one-to-one ratio in terms of technology devices like tablet PCs, Heinrich et al. (2020) suggest that the teachers in such settings could change their approach by encouraging peer collaborative learning as learners share the available devices. This argument supports the social constructivist approach by Vygotsky that emphasizes collaboration as opposed to individual learning (Waweru, 2018). As Sang et al. (2009) explain, teachers in areas without adequate ICT devices need to apply teaching methods that create an environment of cooperation and democracy to enable content sharing among learners. Nonetheless, for this to happen a teacher needs to be equipped with the requisite technology integration skills to be able to assess the learners' use of technology and their use in instruction.

For this reason, we sought to investigate the teachers’ capacity for technology integration in primary schools. The findings pointed to an increase in computer literacy among primary school teachers which has been highlighted as a key determinant in the successful integration of technology in various studies (Hwang et al., 2010; UNESCO, 2012). The results were consistent with previous research which attributed the increase in the number of computer-literate teachers with the introduction of computer courses in the Kenyan teacher training colleges (Omito et al., 2019; Muinde & Mbataru, 2019). However, although computer literacy among teachers is important, it does not guarantee that teachers would use technology in their lessons (Mwangi & Khatete, 2017; Wambiri & Ndani, 2016) because of gaps in the pedagogical application in actual teaching.

Relatedly, we found that most teachers did not integrate ICT in their lessons and had not attended in-service training after the start of the implementation of the new curriculum. This corroborates other studies which concluded that computer literacy training was not enough to guarantee the integration of technology and that teachers needed a deeper understanding of the pedagogical use of ICT (Omito et al., 2019; Ngeno et al., 2020; Sharples & Moldeus, 2014). Further, we found that younger teachers had better technology integration skills compared to older teachers consistent with previous studies which showed that age correlates negatively with skill level in the use of technology (Harrison & Rainer, 1992 cited by Wambiri & Ndani, 2016). However, as noted by Bebell et al. (2004) teachers’ age and years of work may not be conclusive in the measurement of teachers’ technology use. Therefore, a study designed to include a variety of technology uses in schools would give a more detailed account of how teachers interact with technology daily.

Despite the skill gap that exists among teachers in technology integration, our study shows that generally, teachers had a high perception. Similarly, Wambiri and Ndani (2016) concluded that teachers in Kenyan primary schools had high attitudes towards the use of various technologies indicating that with the requisite support the use of ICT in schools would be achieved. This is also supported by the finding that teachers had the high belief that ICT use would not only benefit them in the organization of instruction but also their learners. The perception of the usefulness of technology to learners by teachers is important because it helps the teacher to invoke the innovativeness and creativity of the learner (Kalpana, 2014; KICD, 2017; Wang, 2008; Waweru, 2018). The perception of technology as time-consuming, however, can be attributed to inadequate training on the pedagogical use of ICT as found in previous studies (Sharples & Moldeus, 2014). This means that due to inadequate preparation, such teachers would need the help of computer technicians for successful integration. According to Heinrich et al. (2020), the teachers’ beliefs about time and the effort needed for technology integration generally affect their perception of the ease of use and perceived usefulness to their learners. The perception of learner safety while using the internet could be attributed to inadequate teacher preparation for the safe use to both learners and teachers.

We also analyzed the effect of age and gender on the perception of usefulness and age. Teachers in their 40 s found ICT more useful than their counterparts in the 30 s. This finding was different from previous research that found the perception to be higher among younger teachers (Wambiri & Ndani, 2016). This difference could have been occasioned by sample composition in our study since the number of teachers in the 30 s was two times more than those in the 40 s. However, Bebell et al. (2004) warn that it is not obvious that younger teachers would have a higher perception of technology. A test of how teachers of different ages perceive the usefulness of specific technologies in the performance of their duties would lead to a more detailed analysis. Additionally, our analysis on the effect of gender on the perceived usefulness of technology among teachers did not show any statistical difference. This was consistent with Buliva (2018) who found no significant difference in the perception of technology use among teachers by gender. It, therefore, suggests that exemplary performance in the integration of technology should be expected from all teachers. The results also indicate that policymakers should formulate ways to equip male and female teachers with technology integration skills since they all have high perceptions and significant skill gaps. However, Venkatesh et al. (2003) noted that based on socialization, men would perceive certain technology as more useful if it allowed them to accomplish a task faster.

## Limitations and areas of future research

The sampling schemes can be improved in subsequent research. The online survey combined with convenience sampling was an unavoidable choice at the time of data collection; the Global COVID-19 pandemic led to the closure of schools in Kenya, which may have caused sampling bias and limit the generalizability of the findings. Particularly, only 6% of the respondents were in the age bracket of 50 s, while there were 29% of them in the population. Male teachers were also oversampled in our study. While we had 61% male and 39% female teachers, the proportion in the population was 3:7. We should be cautious in interpreting the findings relating to this class of respondents. Follow-up studies are also recommended to take additional steps to increase validity of the instrument such as obtaining content validity ratio (CVR).

Further, our use of the Technology Acceptance Model (TAM) as the theoretical base of the study could have left out other constructs that would give further understanding of acceptance of ICT. We, therefore, recommend the use of other models such as the United Theory of Acceptance and Use of Technology (UTAUT) in further studies to include other constructs such as social influence and facilitating conditions which would improve the prediction of the intention to use technology.

A replication of this study using a mixed-methods approach would give an in-depth understanding of the issues affecting the implementation of ICT integration in Kenya and other developing countries. More research is needed on the perceptions of technology use among teachers in their 30 s and 40 s as well as the effect of gender on the capacity and perception of teachers. A study on how teachers are using technology for the formative assessment of learners in various subjects would also contribute to accumulating knowledge on the progress of ICT integration in all areas of the curriculum. It would be important to study head teachers’ use of technology in the supervision of curriculum implementation. Future research may also focus on the perception of male and female teachers on the usefulness and ease of use of a specific technology in accomplishing various tasks. Finally, it would be important to do a comparative study between the East African countries since they are in the process of implementing the harmonized curriculum structures and framework for primary education.

## Conclusions

The findings from this study suggest that the ICT facilities were inadequate including laptops for teachers, projectors, tablets PC devices for pupils, as well as other enabling installations. There is a need to provide computers to teachers so that they can easily access materials and prepare for technology integration. This will help to familiarize the teachers with computer hardware and software hence reducing the need for computer technicians in schools.

Secondly, we noted that although most of the teachers had basic computer literacy there was a challenge in technology integration due to inadequate pedagogical knowledge on integration. Teachers implementing the new curriculum should be involved in frequent PD programs and training that goes beyond basic computer literacy to technology integration in various subjects. In circumstances where the shortage of devices is inevitable, teachers should be trained on how to encourage collaboration among learners through the sharing of the technology devices and working on tasks as a team.

The results further indicated that teachers have a high attitude towards the use of ICT regardless of gender and the numerous challenges that they face. To encourage the younger teachers to use technology and to train their older colleagues on integration in teaching, the government should consider giving incentives. A reward such as official recognition or sponsorship for further ICT in education training could act as a good motivator to younger teachers.

## Data Availability

All the data sets are available on request.All the data sets are available on request.
